# Impact of a Novel Two-Phase Natural Deep Eutectic Solvent-Assisted Extraction on the Structural, Functional, and Flavor Properties of Hemp Protein Isolates

**DOI:** 10.3390/plants14020274

**Published:** 2025-01-18

**Authors:** Yi Chen, Wellington S. Oliveira, Fernanda F. G. Dias, Baraem P. Ismail

**Affiliations:** Department of Food Science and Nutrition, University of Minnesota, St. Paul, MN 55108, USA; chen5177@umn.edu (Y.C.); dasilvaw@umn.edu (W.S.O.)

**Keywords:** hemp protein isolate, natural deep eutectic solvents, protein characterization, functional properties, fatty acid composition, flavor

## Abstract

Defatting dehulled hemp seeds is a crucial step prior to protein extraction. However, conventional methods rely on flammable solvents, posing significant health, safety, and environmental concerns. Additionally, hemp protein has poor extractability, challenging functionality, and flavor limitations, restricting its broader application in foods. Accordingly, a two-phase natural deep eutectic solvent (NADES)-assisted extraction was evaluated as a solvent-free alternative for co-extracting protein and oil from full-fat hemp flour. In comparison to the reference hemp protein isolate (R-HPI), produced from hexane-defatted flour following conventional alkaline extraction, NADES-extracted hemp protein isolate (N-HPI) had significantly higher protein extraction yield and purity. N-HPI exhibited enhanced surface charge, lower hydrophobicity, and thus higher solubility at an acidic pH compared to R-HPI. N-HPI had a higher abundance of edestin and lower levels of vicilin-like proteins, which contributed to superior gelation compared to R-HPI. N-HPI, compared to R-HPI, contained lower levels of lipid-derived off-flavor compounds, such as aldehydes, alcohols, and ketones. These findings highlighted, for the first time, the potential of a two-phase NADES-assisted extraction as a sustainable alternate and effective process for producing high-quality, functional hemp protein. The development of such a green process is an impetus for broadening the applications of hemp protein in food systems.

## 1. Introduction

Increasing consumer desire for high-protein, plant-based food products is driving the demand for novel protein sources. Consequently, the plant protein market is projected to reach USD 19.2 billion by 2028 with a compound annual growth rate (CAGR) of 7.7% from 2023 to 2028 [1]. The increasing interest in plant proteins is attributed to the health benefits of plant-based diets, concerns for animal welfare, and interest in environmental sustainability [2,3]. The rapid growth in the plant-based market has spurred the development and improvement in processing technologies to promote sustainable protein production.

Prominent plant protein sources such as soy, wheat, and pea face challenges that impact their use in food systems. Soy protein, while highly functional and nutritious, raises consumer concerns because of its allergenicity and status as a genetically modified organism (GMO). Gluten is also avoided by consumers with gluten allergenicity or sensitivities, and by consumers with celiac disease [4]. Pea protein, which is nonallergen and non-GMO, emerged as a strong contender, yet is inferior to soy protein in functional properties [5]. These limitations underscore the need for the exploration of novel protein sources, such as hemp oilseed.

Hemp oilseeds have great potential as a valuable source of food ingredients, including high-quality plant protein and oil suitable for both food and industrial applications [6]. However, the study of hemp oilseeds as a food source has been limited due to legal restrictions related to its classification within the same species, *Cannabis sativa*, as marijuana [7]. While hemp and marijuana differ significantly in their chemical composition and uses, their close botanical relationship has historically led to regulatory challenges. In 2018, these restrictions on hemp cultivation and food use in the US were lifted, paving the way for the incorporation of hemp ingredients in food products. Moreover, the Food and Drug Administration (FDA) granted GRAS (Generally Recognized as Safe) status for three commonly sold hemp seed-derived food ingredients: hulled hemp seeds, hemp seed protein powder, and hemp seed oil [8].

Hemp seed-derived food ingredients, such as protein powders, require extensive upstream processing. Effective protein extraction requires initial lipid removal, typically achieved through mechanical pressing and solvent extraction [9,10,11]. Although mechanical pressing yields high-quality oil, its extraction efficiency is low, and the resulting protein-rich press cake has a high residual oil content [9]. Therefore, the press cake is often subjected to solvent extraction to remove the residual oil and obtain a defatted meal that can be milled into flour. The hemp flour has a relatively high protein content (50–60%) and can be used to produce a protein isolate (>80% protein) [10,11,12].

Although hexane is considered an efficient and cost-effective solvent for oil removal, its use raises concerns related to human health and environmental safety [13]. On the other hand, the production of protein isolate from the defatted flour requires the adoption of harsh alkaline conditions (pH > 10) to extract the protein [12]. High alkalinity results in protein denaturation and aggregation, leading to detrimental effects on the functional behavior of the protein [12]. In addition, high alkalinity during plant protein processing has been associated with the development of off-flavors in pulses, such as pea protein [14,15]. However, similar studies on oilseeds are currently lacking. Therefore, to preserve functional properties and reduce potential off-flavor development, it is necessary to develop novel, sustainable, and green alternative processes to produce high-quality plant proteins from oilseeds, such as hemp oilseeds.

In this context, natural deep eutectic solvents (NADES) have recently drawn attention as innovative and versatile green solvents due to their eco-friendliness and biodegradability [16]. These solvents are composed of naturally occurring, nontoxic substances such as quaternary ammonium salts, polyols, sugars, amino acids and organic acids, suitable for food applications [16,17]. NADES also possess several advantageous properties such as low vapor pressure, high thermal stability, adjustable viscosity, and excellent miscibility with a wide range of organic and inorganic compounds [17]. Recent research has demonstrated that NADES can be used to extract protein under mild conditions and improve the functionality of protein isolates obtained from the defatted meals of canola, pomegranate, flaxseed, and sunflower seeds, among others [18,19,20]. The mechanism involved in protein extraction using NADES depends on the formation of a robust hydrogen-bonding network that effectively penetrates cellular matrices and disrupts hydrophobic interactions [21,22]. In addition, the ionic and electrostatic interactions within NADES alter the protein’s microenvironment, reducing protein–protein interaction and enhancing solubility and extractability [23]. However, a significant drawback of current NADES utilization is its dependence on an upstream hexane defatting step, highlighting the need for developing sustainable approaches that eliminate the need for an organic solvent and simultaneously co-extract both oil and protein.

Aqueous and enzyme-assisted extraction processes were explored for the simultaneous extraction of oil and protein from several full-fat matrices, including almonds [24], chickpeas [25], and coffee [26]. However, such processes were challenged by their low extractability yields, and by the formation of an emulsion phase. This emulsion phase needed to be broken down to allow oil recovery [27,28]. In addition, the use of enzymes resulted in significant protein hydrolysis that could be detrimental to functionality and taste [29,30]. In this context, the development and application of NADES-assisted co-extraction of protein and oil represents a promising solution. Although not yet explored, NADES can be applied in a two-phase system to facilitate the simultaneous extraction of protein and oil from oil-bearing materials. This approach has the potential to improve extraction yields, promote phase separation, and break down emulsions, addressing key limitations associated with traditional aqueous extraction methods. Developing NADES-assisted extraction techniques for the simultaneous extraction of protein and oil could significantly advance the efficiency of green extraction and open new possibilities for food applications.

This work aimed at assessing, for the first time, the effects of a solvent-free process to co-extract protein and oil, from full-fat hemp seeds. Specifically, the objective of this research was to assess the effects of NADES-assisted extraction on hemp protein extractability, purity, composition, structural, and functional properties, and flavor characteristics. Hemp protein isolate extracted from defatted hemp meal following alkaline-assisted extraction coupled with isoelectric precipitation was used as a reference. Commercial proteins (soy and pea protein isolates) were also evaluated for comparison. The findings of this work will provide essential insights into the potential of NADES-assisted extraction as a sustainable alternative for producing high-quality hemp protein for food applications.

## 2. Materials and Methods

### 2.1. Samples and Materials

Hemp seeds from the commercial variety X-59 were dehulled and milled into full-fat hemp flour by Hemp Acres (Waconia, MN, USA). Commercial soy protein isolate (cSPI, ProFam 974, 90.4% protein) and commercial pea protein isolate (cPPI, ProFam 580, 75.8% protein) were acquired from Archer Daniels Midland (ADM) (Decatur, IL, USA). SnakeSkinTM dialysis tubing (3.5 kDa cut off), Imperial™ Protein Stain, and Sudan Red 7B were purchased from Thermo Fisher ScientificTM (Waltham, MA, USA). 8-anilino-1-napthalenesulfonic acid ammonium salt (ANS), Costar^®^ 96-well black opaque plates, butylated hydroxytoluene (BHT), n-alkanes ladder, ethylenediaminetetraacetic acid disodium salt dihydrate (EDTA2Na), HPLC-grade hexane, and chloroform, were sourced were from Sigma-Aldrich (St. Louis, MO, USA). Choline chloride (ChCl), glycerol (Gly), menthol, and thymol were also purchased from Sigma-Aldrich. Criterion™ TGX™ 4–20% precast gels, Laemmli sample buffer, 10× Tris/Glycine/sodium dodecyl sulfate (SDS) running buffer, and Precision Plus molecular weight marker were purchased from Bio-Rad Laboratories, Inc. (Hercules, CA, USA). Folded capillary tubes used for the determination of zeta potential were obtained from Malvern (Malvern, UK). Chromatographic columns, free fatty acid phase FFAP columns (30 m × 0.25 mm × 0.25 µm) and DB-5ms Ultra Inert columns (30 m × 250 μm × 0.25 μm), were purchased from Agilent Technologies Inc. (Santa Clara, CA, USA). Triheptadecanoin (Tri-C17:0) was obtained from Cayman (Ann Arbor, MI, USA). Fatty acid methyl ester (FAME) 37 mix standard was purchased from Supelco (Bellefonte, PA, USA). Hexanal-d5, methylpyrazine-d6, hexyl alcohol-d13, and heptanone-d5 were acquired from CDN Isotopes Inc. (Pointe-Claire, QC, Canada). All other analytical-grade reagents were purchased from Thermo Fisher Scientific or Sigma-Aldrich.

### 2.2. Natural Deep Eutectic Solvent (NADES)-Assisted Extraction

#### 2.2.1. Preparation of NADES

Hydrophilic and hydrophobic NADES solvents were prepared by mixing ChCl and Gly, and menthol and thymol at a 1:1 molar ratio, respectively. The NADES mixtures were heated to 80 °C and stirred at 500 rpm until a clear solution was obtained. The solutions were cooled to room temperature and water was added to the hydrophilic NADES at 40% (w/w).

#### 2.2.2. Oil and Protein Co-Extraction

To determine the optimal NADES-assisted extraction conditions, the impact of hydrophilic to hydrophobic solvent ratio on protein extraction efficiency was assessed by measuring protein yield and purity. In triplicate, full-fat hemp flour (5 g) was dispersed in 100 g of hydrophilic solvent (5% total solids) with 0 g (100:0), 5 g (100:5), or 15 g (100:15) of hydrophobic solvent. The pH was adjusted to 7 and the slurry was agitated for 1 h at room temperature. The dispersion was centrifuged at 12,000× *g* for 15 min. The fiber-rich pellet and the gums fraction right above the pellet (containing residual protein, lipids, and carbohydrates not extracted in the process) were collected individually and redispersed in double distilled water (DDW, 10% total solids), dialyzed against water, and lyophilized. The supernatant was poured into a separatory funnel to allow for the separation of the protein-rich and oil-rich phases overnight. The protein-rich solution was collected, dialyzed against water, and lyophilized. The oil layer was collected, and the separatory funnel was rinsed with a small amount of hexane to ensure proper oil recovery, which was then evaporated under nitrogen flux. All fractions were weighed for mass balance evaluation. To determine protein yield and purity, the protein content of each fraction was measured following the Dumas AOAC Method 990.03 using a LECO nitrogen analyzer (St. Joseph, MI, USA) and a protein conversion factor of 5.30 [31]. Fat content was analyzed following the Mojonnier AOAC method 922.06 [32]. Moisture content was determined following the vacuum oven AACCI method 44–40.01 [33]. Ash content was measured following the dry ashing AOAC method 923.03 [32]. NADES-assisted extraction was repeated under the conditions that had the best yield and purity (at hydrophilic and hydrophobic solvent ratio of 100:5 g) to produce a sufficient amount of NADES-extracted hemp protein isolate (N-HPI) for structural, functional, and flavor characterization.

### 2.3. Alkaline-Assisted Extraction Coupled with Isoelectric Precipitation (AE-IEP)

#### 2.3.1. Preparation of Defatted Hemp Meal (DHM)

Full-fat hemp flour was defatted using hexane following the procedure outlined by Eckhardt et al. [12]. with no modification. The defatted hemp meal (DHM) was milled to 50-mesh using a cyclone sample mill (UDY Corp, Fort Collins, CO, USA). The milled DHM had a fat content of 1.5% on a wet basis (w.b.) as assessed by the Mojonnier method. The protein content was 62.5% w.b. as determined by the Dumas method.

#### 2.3.2. Protein Extraction

Hemp protein isolates were produced from DHM following the AE-IEP described by Eckhardt et al. [12]. The initial protein solubilization was performed at pH 7, 8, 9, 10, and 11 (Supplementary Material—Table S1) to select the extraction pH for the reference pH-extracted hemp protein isolate (R-HPI). In triplicate, DHM (5 g) was dispersed in 100 mL of DDW (5% w/v), and the pH was adjusted to 7, 8, 9, 10, or 11 with 2 N NaOH. The dispersion was stirred for 1 h and centrifuged at 12,000× *g* for 15 min. The supernatant was collected and neutralized. The pellet was redispersed in DDW (5% w/v) for a second round of solubilization at pH 7, 8, 9, 10, or 11, followed by stirring for another hour. After centrifugation, the supernatant was collected, neutralized, and combined with the first supernatant. The residual pellet was retained and lyophilized. The combined supernatant was adjusted to a precipitation pH of 5, followed by centrifugation. The supernatant was collected and lyophilized. The protein pellet was dispersed in DDW (1:4 w/w) and neutralized before dialysis and lyophilization. All lyophilized fractions were weighed for mass balance determination. The protein yield and purity were determined by measuring the protein content of each fraction following the Dumas method. Fat, moisture and ash contents were determined as described in Section 2.2.2.

#### 2.3.3. Evaluation of Protein Solubility

The protein solubility of the HPI samples produced at different solubilization pHs (pH 7, 8, 9, 10, 11) determined following the method outlined by Boyle et al. [34] and modified by Bu et al. [35]. Protein solubility was assessed, in triplicate, at 5% (w/v) protein concentration, at pH 7 and pH 3.4, with and without heating at 80 °C for 30 min. Based on protein yield and purity (Supplementary Material—Table S1), and protein solubility results (Supplementary Material—Table S2), the extraction pH of the R-HPI was determined to be pH 8. A sufficient amount of R-HPI was produced (pH 8 extraction) for structural, functional, and flavor characterization.

### 2.4. Color Measurement

The color of R-HPI, N-HPI, cSPI, and cPPI samples was measured, in triplicate, following the CIE (International Commission on Illumination) 1976 L* a* b* color system using a Chroma Meter CR-221 (Minolta Camera Co., Osaka, Japan), as outlined by Eckhardt et al. [12]. with no modification. L* represents lightness ranging from 0 (black) to 100 (white); a* denotes the red–green axis with positive values indicating redness and negative values indicating greenness; b* corresponds to the yellow–blue axis with positive values for yellowness and negative values for blueness.

### 2.5. Protein Structural Properties

#### 2.5.1. Protein Profiling by Gel Electrophoresis

The protein profile of R-HPI and N-HPI was monitored using sodium dodecyl polyacrylamide gel electrophoresis (SDS-PAGE) following the method described by Boyle et al. [34]. The protein samples were dispersed in DDW (20 mg protein/mL) and solubilized for 2 h. The solubilized samples were prepared under non-reducing (only Laemmli buffer) and reducing (Laemmli buffer with β-mercaptoethanol (βME) conditions. Protein samples (50 µg protein) and Plus™ MW standard were loaded onto a 4–20% precast gradient gel. The gel was electrophoresed, stained, destained, and imaged (Molecular Imager Gel Doc XR system, Bio-Rad Laboratories), as previously reported.

#### 2.5.2. Protein Surface Properties

Surface hydrophobicity and Zeta potential of R-HPI and N-HPI were measured, in triplicate, using the ANS spectrofluorometric method and the dynamic light scattering instrument, Malvern Nano Z-S Zetasizer, respectively, as outlined by Bu et al. [35] and modified by Eckhardt et al. [12].

#### 2.5.3. Thermal Denaturation by Differential Scanning Calorimetry (DSC)

Denaturation temperature and enthalpy of the protein in R-HPI and N-HPI were determined, in triplicate, using a DSC instrument (DSC 1 STARe System, Mettler Toledo, Columbus, OH, USA), based on the method outlined by Eckhardt et al. [12], with no modification. Thermograms were collected and analyzed using Mettler Toledo’s STARe Software version 11.00 to determine the peak denaturation temperature and enthalpy for each protein.

### 2.6. Protein Functional Properties

#### 2.6.1. Protein Solubility

The protein solubility of N-HPI, R-HPI, and commercial samples was measured as described in Section 2.3.3.

#### 2.6.2. Emulsification Capacity

The emulsification capacity (EC) of R-HPI and N-HPI was determined, in triplicate, as described by Bu et al. [35]. In brief, protein solutions at 1% (w/v) concentration were homogenized using an IKA^®^ RW 20 (IKA Works Inc., Wilmington, NC, USA) Digital mixer equipped with a four-blade, 50 mm diameter shaft (IKA^®^ R 1342) rotating at 860–870 rpm. Corn oil colored with Sudan Red 7B was gradually titrated at a steady flow rate into the protein solution while homogenizing. The addition of oil continued until phase inversion occurred. EC was calculated as the amount of oil (in grams) emulsified per gram of protein.

#### 2.6.3. Gel Strength and Morphology

Protein solutions at concentrations of 15% or 20% (w/v in DDW) were prepared in triplicate, adjusted to pH 7, and stirred for 2 h to ensure adequate dispersion. The protein solutions (1 mL aliquots) were heated at 95 °C (±2 °C) in a water bath for 15 (cSPI) or 20 min (cPPI, R-HPI, N-HPI) and cooled to room temperature. Gel strength was measured using a TA-XT Plus Texture Analyzer (Stable Micro Systems LTD, Surrey, UK) with a 100 mm diameter probe, a 5 mm/s test speed, and a target distance of 0.5 mm from the plate. The force in Newtons (N) required to rupture each gel was recorded as gel strength. Scanning electron microscopy (SEM) was used to visualize the morphology of the lyophilized protein gels. The gels were sliced horizontally into 1 cm sections using a razor blade and were affixed onto aluminum stubs with double-sided carbon adhesive tabs. The samples were sputter-coated with 60/40 gold–palladium mixture. Scans were performed using a Hitachi S-3500N SEM (Hitachi, Tokyo, Japan) at an accelerating voltage of 5 kV. Cross-sectional images were captured at 500× and 1000× magnification.

### 2.7. Impact of Residual Fat on Off-Flavor Development

#### 2.7.1. Fatty Acid Profile

Fatty acid profile of hemp flour (HF), N-HPI, R-HPI, cSPI, and cPPI was determined as described by Dias et al., [36]. Briefly, sample aliquots containing ~4 mg of fat were weighed, in triplicate, into glass tubes. Lipids were extracted using the Folch extraction method and trans-esterified using methanolic HCl to generate fatty acid methyl esters (FAME). The lipid extracts were spiked with 0.6 mg of the internal standard triheptadecanoin (Tri-C17:0, 15 mg/mL). The samples were analyzed using a gas chromatograph coupled with a flame ionization detector (GC-FID, Agilent 6890N, Agilent Technologies, Santa Clara, CA, USA). One microliter of each sample was injected in split mode (1:30). Separation was achieved using an FFAP column (30 m × 0.25 mm × 0.25 µm). The injector was set at 240 °C, and the detector at 300 °C. The oven temperature program was set at 50 °C for the first 2 min, increased to 180 °C at a rate of 10 °C/min, then ramped up to 240 °C at 5 °C/min, and was maintained at 240 °C for 13 min. Hydrogen was used as the carrier gas at a flow rate of 1 mL/min. Peak identification was carried out by comparing the retention times of the FAME 37 standards with those of the samples under the same conditions. Peak identification and integration were performed using Chemstation software B.04.03 (Agilent Technologies, Inc., Santa Clara, CA, USA). Relative quantification was performed using the internal standard.

#### 2.7.2. Profile of Volatile Organic Compounds

Volatile organic compounds were extracted, in triplicate, from HF, N-HPI, R-HPI, cSPI, and cPPI following a solid-phase microextraction (SPME), using A 2 cm Divinylbenzene/Carboxen/Polydimethylsiloxane (DVB/CAR/PDMS) fiber as described by Oliveira et al. [37]. Analyses were conducted using an Agilent 6890 gas chromatograph, equipped with a PAL RSI 120 autosampler and an Agilent 5973 single quadrupole mass spectrometer (Agilent Technologies, Palo Alto, CA, USA) and a DB-5ms Ultra Inert column (30 m × 250 μm × 0.25 μm). Volatile organic compounds were identified using mass spectrometry (MS) with MassHunter Workstation Unknown analysis software version 12.0.893.1 (Agilent Technologies, Inc., Santa Clara, CA, USA) and the NIST 17 MS library. Compounds were identified if they had a signal-to-noise ratio greater than 3 and a match score above 80. To confirm the identities of the compounds, programmed temperature retention indexes (RIs) were calculated according to Van den Dool and Kratz methods by analyzing a solution of n-alkanes (C7–C20) under the same conditions. Relative quantification was carried out according to Xiao et al. [38] using four deuterated internal standards from different chemical classes (hexanal-d5, methylpyrazine-d6, hexyl alcohol-d13, and heptanone-d5), each at a concentration of 0.5 mg/kg. The relative concentration was used to compare the profiles among the samples.

### 2.8. Statistical Analysis

IBM^®^ SPSS^®^ Statistics software version 28 for Windows (International Business Machines Corp., Armonk, NY, USA) or Statistica 14 software (Statsoft, Tulsa, OK, USA) was used for one-way analysis of variance (ANOVA). Tukey–Kramer multiple means comparison test was used to determine the significant differences (*p* ≤ 0.05) among the means of three or more samples. Independent-samples *t*-test was used to determine significant differences (*p* ≤ 0.05) between the average of two samples. Principal component analysis (PCA) was performed to evaluate the differences among the variables and track trends among the samples. The data were auto-scaled and analyzed using MetaboAnalyst 5.0 “https://www.metaboanalyst.ca (accessed on 10 December 2024)”. The PCA was performed using the concentration of each identified volatile compound.

## 3. Results and Discussion

### 3.1. Effects of NADES-Assisted Extraction on Protein Extractability and Composition

Given that the NADES-assisted extraction process was conducted at pH 7.0 and that hemp protein generally requires high alkaline pH for optimal extraction [12], a series of preliminary tests were carried out to identify alkaline-assisted extraction conditions best suited to produce a sufficient amount of R-HPI for comparison with N-HPI. The protein extraction at pH 7.0 (the same as the pH used for NADES-assisted extraction) resulted in a very low protein extraction yield (Supplementary Material—Table S1). Such a low yield was insufficient and impractical for the production of enough protein isolate needed for the characterization assays, prompting trials at higher pH levels. Protein extraction yields and purity significantly increased as the extraction pH increased from 7 to 11 (Supplementary Material—Table S1).

Protein solubility was also notably influenced by the extraction pH. Protein solubility tested at pH 7 was the highest for the HPI extracted at pH 7, followed by that of the HPI extracted at pH 8.0 (Supplementary Material—Table S2). As previously reported, high alkalinity during protein extraction resulted in a very low protein solubility, which was attributed to excessive protein denaturation and polymerization [12,39,40,41]. Under acidic conditions (pH 3.4), protein solubility was comparable among the samples, with minor statistical differences (Supplementary Material—Table S2). Considering these solubility results and protein yields, pH 8 was chosen as the reference extraction pH for producing R-HPI, under relatively mild extraction conditions.

NADES-assisted extraction, under varying ratios of hydrophilic and hydrophobic solvents, was effective in achieving high protein extraction yields and high protein purities (Table 1). Most importantly, the N-HPI samples had low residual lipid and low ash content (Table 1), demonstrating the success of this approach in co-extracting oil and protein from full-fat hemp flour, without the need for flammable solvents. It is also important to highlight that after membrane filtration (dialysis step) the purified protein is free from residual NADES. Moreover, the use of low amounts of non-polar NADES (100 g of hydrophilic + 5 g of hydrophobic) contributed to the highest protein purity (Table 1) and a significantly lower fat content compared to the NADES with no hydrophobic solvent. This observation demonstrated, for the first time, that the addition of the hydrophobic NADES enhanced phase separation between the oil-rich phase and the protein-rich phase. The successful use of two-phase NADES for the co-extraction of oil and protein from the full-fat material is unique and has not been previously reported.

NADES-assisted extraction had a protein extraction yield 10-fold higher than that of pH 8 reference extraction (Table 1 and Supplementary Material—Table S1). In fact, NADES-assisted extraction, performed at pH 7, had a comparable protein yield and purity (Table 1) to the pH extraction performed at pH 11 (Supplementary Material—Table S1). This observation confirmed the remarkable advantage of using NADES to achieve high protein extraction yield and purity, without the need for prior defatting with organic solvent and for harsh alkalinity during the extraction. In contrast to the harsh alkalinity, the mild conditions of the NADES-assisted extraction would most likely preserve the protein’s native structure and prevent the impairment of its functionality.

Water is a highly polar solvent, which is not ideal for extracting highly hydrophobic proteins, such as many plant proteins [42]. However, in a highly alkaline aqueous medium, many of the protein side chains become charged, increasing protein–water interaction and protein solubility, leading to enhanced extractability [43]. In comparison, the effectiveness of hydrophilic NADES in extracting plant protein is attributed to being less polar than water. Hydrophilic NADES offer a combination of polar and non-polar functional groups, which in turn enable them to solubilize plant proteins by forming various types of non-covalent interactions with the protein’s complex structure.

A similar observation of high protein extraction yield was reported when using NADES composed of choline chloride and glycerol in comparison with alkaline (pH 9.5) protein extraction from fava bean (<2% fat) [44]. High protein extraction yields were also reported for defatted canola meal (<1% fat) when using NADES composed of choline chloride and urea in comparison to alkaline extraction at pH 9 [18]. While alkaline extraction at pH 12 led to a higher canola protein extraction yield, the authors highlighted that the use of NADES led to better preservation of the native protein structure compared to alkaline extraction. Nevertheless, there are no reports on utilizing one-phase or two-phase NADES extraction from full-fat matrices.

Given the promising results of high protein extractability, purity, and low-fat content, two-phase NADES (100 g of hydrophilic + 5 g of hydrophobic) was selected to produce N-HPI for further characterization. To prove the positive impact of the two-phase NADES-assisted extraction on hemp protein characteristics, N-HPI was compared to R-HPI and commercial protein isolates (cSPI and cPPI).

### 3.2. Effects of NADES-Assisted Extraction on the Color of HPI

N-HPI was the lightest in color and the least yellow, having the significantly highest L* value and the lowest b* value, respectively, among all the samples evaluated (Table 2). In contrast, R-HPI was the darkest in color among the samples, having the significantly lowest L* value. The darker color in R-HPI compared to N-HPI, could be attributed to oxidation of phenolics under alkaline conditions [12]. The lighter color of N-HPI compared to the commercial isolates (cSPI and cPPI), provided another positive edge. Protein ingredients that are light in color are preferred in food applications to avoid undesirable colors in the final product. Visual appeal and color stability are essential for consumer acceptance.

Similarly, Karimi et al. [18] reported that the NADES-extracted canola protein had a lighter and improved color when compared with the alkaline-extracted proteins. The authors attributed the dark color of the alkaline extracted proteins to the formation of quinones from phenolic compounds under alkaline conditions and their subsequent interactions with either nucleophile amino acids or other phenolic compounds.

### 3.3. Effects of NADES-Assisted Extraction on the Protein Structural Properties

#### 3.3.1. Protein Profile

**Figure 1:**
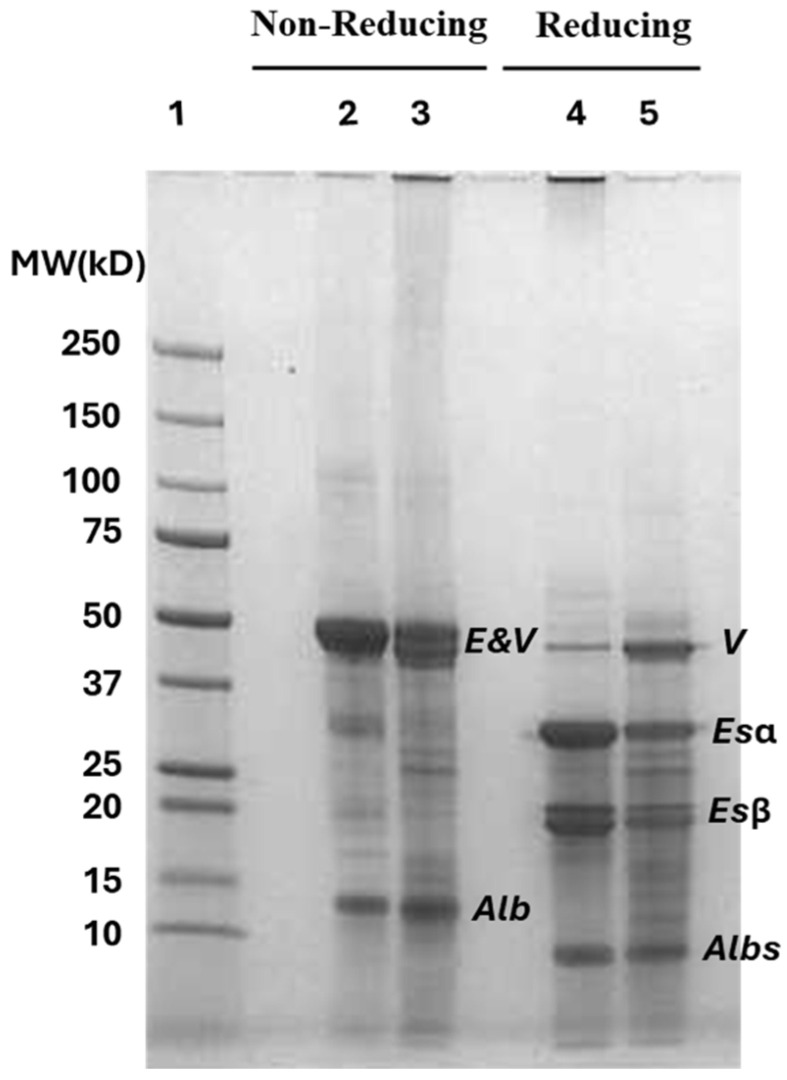
SDS-PAGE gel protein profile visualization of NADES-extracted and reference pH-extracted hemp protein isolates (N-HPI and R-HPI) under non-reducing and reducing conditions. Lane 1: Molecular weight (MW) marker; Lane 2, 3: non-reduced N-HPI and R-HPI; Lane 4, 5: reduced N-HPI and R-HPI. E: 11S edestin monomer; V: 7S vicilin-like protein monomer; Alb: 2S albumin; Esα: acidic subunit cleaved from edestin monomer; Esβ: basic subunit cleaved from edestin monomer; Albs: albumin subunits.

Two major differences were noted in the protein profiles of the two samples, N-HPI and R-HPI. Under non-reducing conditions, N-HPI had a more prominent 11S edestin monomer at 50 kDa and a less prominent 7S vicilin-like protein compared to R-HPI (Figure 1). The vicilin-like protein has a close molecular weight to edestin monomer and can be more distinguished under reducing conditions, where edestin is reduced to its α and β subunits that are typically bonded by disulfide linkages [12]. The vicilin-like protein has no disulfide linkages; thus, it appeared at around 50 kDa under both non-reducing and reducing conditions [12]. This observation indicated that NADES-assisted extraction favored the more hydrophobic edestin, while mild alkaline extraction favored the less hydrophobic vicilin-like protein. Given the high hydrophobicity of edestin, it requires high alkalinity that imparts negative charges and promotes the solubility of edestin. The extraction of R-HPI was at a mild alkalinity, of pH 8, not sufficient to effectively solubilize edestin.

The other noticeable difference is the longitudinal smearing at the upper part of the gel for the R-HPI under non-reducing conditions, which was not apparent for N-HPI (Figure 1, lane 3 compared to lane 2). This observation confirmed that the alkaline extraction, even at a mild pH, enhanced disulfide linkages, resulting in the formation of soluble protein polymers of wide molecular weight distribution. The smearing disappeared in the R-HPI lane under reducing conditions (Figure 1, Lane 5), due to the reduction of disulfide bonds. In contrast, high molecular weight bands appeared at the top of the N-HPI lane under reducing conditions (Figure 1, lane 4). This observation indicated that the relatively high content of edestin in N-HPI could have contributed to the formation of large insoluble aggregates, stabilized by disulfide bonds, and could not be seen under non-reducing conditions. These aggregates could have precipitated before loading the sample on the gel under non-reducing conditions. Adding a reducing agent potentially broke down these polymers enough to allow them to stay in the solution and migrate down the gel.

NADES salts were dialyzed out of the sample before lyophilization. The removal of these salts will contribute to the reduced solubility of the hemp protein, especially edestin at pH 7. These notable protein profile differences between N-HPI and R-HPI will have a noted impact on protein functionality.

#### 3.3.2. Protein Surface Properties

Surface hydrophobicity impacts the proteins’ intermolecular interactions with each other, with water, and/or oil. Therefore, surface hydrophobicity has a direct bearing on protein solubility, emulsification, and gelation [45,46].

Both N-HPI and R-HPI samples, which were neutralized before drying, had significantly higher surface hydrophobicity (measured at pH 7) than cSPI and cPPI (Table 3). Similar results were previously reported by Eckhardt et al. [12] who also found higher surface hydrophobicity for HPI samples in comparison with cSPI and cPPI. Such high surface hydrophobicity at pH 7 may negatively impact protein solubility, as it limits the ability of the protein to interact with water. In general, hemp protein has low extractability and solubility at pH 7 (Supplementary Material—Tables S1 and S2). Such high surface hydrophobicity limits protein–water interactions and drives protein–protein interactions via attractive hydrophobic forces. When in close proximity, disulfide interactions are made possible, explaining the observed protein polymerization in both samples (Figure 1).

However, the surface hydrophobicity of HPI reported by Eckhardt et al. [12] was 1.5 times higher than the observed surface hydrophobicity of R-HPI and N-HPI. This observation confirmed that N-HPI did not cause as much unfolding as an alkaline extraction performed at a very high pH (pH 11). This difference in the impact of extraction conditions on the protein’s surface hydrophobicity may have a rather positive consequence on the protein’s functionality.

Surface charge is another key factor that influences intermolecular interactions. At pH 7.0, both R-HPI and N-HPI had relatively lower net charges than both cSPI and cPPI (Table 3). N-HPI had a slightly lower net charge than R-HPI, which could be attributed to its higher abundance of edestin (Figure 1). In contrast, N-HPI had a significantly higher net charge at pH 3.4. Again, this is attributed to the higher abundance of edestin in N-HPI than in R-HPI. Edestin has a higher isoelectric point than vicilin-like protein; thus, edestin will carry a higher net charge at 3.4, farther away from its isoelectric point. Even though N-HPI and R-HPI have similar surface hydrophobicity, it is anticipated that N-HPI will have higher solubility than R-HPI at pH 3.4.

#### 3.3.3. Protein Thermal Denaturation

DSC provides the thermal properties of proteins including temperature of denaturation (Td) and enthalpy (ΔH). Td reflects the thermal stability of the proteins, while ΔH reflects the proportion of undenatured proteins as well as the extent of ordered structure [43]. The commercial samples (cSPI and cPPI) had no endothermic peaks, indicating complete protein denaturation (Table 3), in accordance with previous reports [12,47]. In contrast, both N-HPI and R-HPI showed endothermic peaks, indicating that these proteins were not completely denatured during the extraction process (Table 3). The protein Td of both HPI samples was within the previously reported range of 77–95 °C [12,48]. The ΔH for R-HPI fell within the reported range of 5–12 J g^−1^ for alkaline-extracted HPI [12,48], while N-HPI exhibited a significantly higher ΔH (Table 3). This observation is mostly attributed to the higher abundance of edestin in N-HPI than in R-HPI. Edestin is stabilized by disulfide bonds, while vicilin-like protein is not. Proteins stabilized by disulfide bonds require higher energy to denature them than those stabilized by non-covalent interactions [49]. Nevertheless, results confirmed that both extractions were mild and did not cause significant protein denaturation, unlike extractions performed at relatively high alkalinity (pH 11) [12].

### 3.4. Effects of NADES-Assisted Extraction on the Protein Functional Properties

#### 3.4.1. Protein Solubility

Protein solubility of N-HPI and R-HPI was assessed at pH 3.4 and 7, both under non-heated and heated conditions (Table 4), to evaluate their suitability for use in acidic and neutral beverage formulations. The solubility of N-HPI and R-HPI was evaluated against commercial protein ingredients (cSPI and cPPI) to determine the potential of replacing these ingredients in the protein beverage market.

Both N-HPI and R-HPI had inferior solubility compared to cSPI at pH 7. This observation, which was consistent with previous reports [12,47,50,51], was attributed mostly to the more than double surface hydrophobicity of HPI samples and about half the surface charge at pH 7, compared to Cspi (Table 3). Accordingly, replacing cSPI in neutral beverages is a far fetch for these HPI samples. R-HPI performed better than N-HPI at pH 7 due mostly to the lower abundance of edestin in R-HPI. As discussed, a higher abundance of edestin in N-HPI compared to R-HPI resulted in a significantly lower surface charge (Table 3) and the presence of insoluble aggregates at pH 7 (Figure 1). In addition, 7S vicilin-like protein forms smaller molecular weight quaternary structures than 11S edestin, contributing to higher solubility at pH 7 [52]. The protein solubility of R-HPI considerably surpassed that of HPI extracted at pH 11 [12], even when protein solubility was measured at only 1% protein concentration versus the 5% protein concentration used in this study. As discussed, the high alkalinity used by Eckhardt et al. [12] to extract the protein resulted in protein denaturation and aggregation, leading to their reported poor solubility.

In contrast, N-HPI outperformed R-HPI and the commercial samples at pH 3.4 with solubility exceeding 90% under both non-heated and heated conditions (Table 4). As discussed, the relatively high proportion of edestin in N-HPI contributed to a high surface charge at pH 3.4 (Table 3), which in turn caused enhanced protein–water interactions. The high Td and ΔH of the HPI samples (Table 3) compared to commercial isolates contributed to their stability even after heating. Moreover, the lower ash content in N-HPI compared to R-HPI (Table 1 and Table S1) could have contributed further to the difference in their protein solubility. The additional salt in R-HPI could have shielded some of the charges on the protein and competed for water, thereby hindering protein–water interactions [47]. Notably, at pH 3.4 N-HPI outperformed considerably the HPI extracted at pH 11 [12], potentially due to lower surface hydrophobicity and higher enthalpy, further confirming the positive impact of the mild NADES-assisted extraction.

The very low protein solubility of the commercial ingredients at pH 3.4 has been observed previously [50]. Such low solubility was attributed to the relatively harsh alkaline extraction conditions that caused excessive denaturation and polymerization, to the low net charge at a pH close to their isoelectric point, and to the relatively high ash content (4–5%). The protein solubility in this study was measured at 5% protein concentration, which is above the minimum requirement (4.2%) for a high protein claim. This observation highlights the potential of N-HPI for use in high-protein acidic beverages replacing and outperforming both soy and pea protein. Further, this high solubility will allow for broader applications in acidic environments, making N-HPI particularly suitable for food and beverage systems that require stable protein solutions under acidic conditions.

#### 3.4.2. Emulsification Capacity

N-HPI had significantly lower EC than R-HPI (Table 4). This observation could be attributed to the notably lower surface charge at pH 7 and the resulting lower solubility of N-HPI compared to R-HPI (Table 3 and Table 4). The higher abundance of edestin in N-HPI could have also contributed to low structural flexibility and slow migration to the interface due to the high molecular weight, disulfide linkages, and compact structure [53,54]. In addition, the surface charge of N-HPI impacted its solubility in the aqueous phase, with lower solubility potentially causing slower diffusion rates to the interface [55,56]. 7S vicilin-like proteins are known to be better emulsifiers than 11S legumins, due to a better balance between attractive and repulsive forces, smaller molecular weight, higher flexibility (due to lack of disulfide linkages), and higher solubility. R-HPI was apparently higher in 7S vicilin-like protein and lower in 11S edestin (Figure 1). The EC of R-HPI was, accordingly, comparable to that of cPPI, but remained inferior to that of cSPI. The performance of cSPI was consistent with previous reports [12,47,51]. Due to its advantageous balance between surface hydrophobicity and surface charge, cSPI outperformed cPPI, which had lower surface charge, higher surface hydrophobicity, and lower solubility at pH 7 (Table 3 and Table 4). Although N-HPI had lower EC than the rest of the samples, it performed better than the HPI extracted at pH 11, which did not form emulsions at all [12]. Again, harsh alkaline extraction conditions proved to be detrimental to protein functionality in comparison to mild NADES-assisted extraction.

#### 3.4.3. Gel Strength and Morphology

The gel strength of N-HPI and R-HPI was assessed at 15% protein concentration in water to determine their potential contribution to the texture and structural integrity of food products such as meat and dairy alternatives. Their performance was compared to that of cSPI (15% protein) and cPPI (20% protein).

Remarkably, N-HPI outperformed R-HPI (Table 4) by far and the two commercial isolates, including cSPI, which is known as a strong gelling agent among plant proteins [47]. This observation is mostly attributed to the higher abundance of edestin. As discussed, edestin forms large molecular-weight polymers, is abundant in sulfhydryl groups, and has a high surface hydrophobicity. These characteristics appeared to be favorable for gel formation and gel strength. In addition, the relatively low solubility of N-HPI (Table 4) is favorable for protein–protein interactions, contributing to the formation of a strong and stable three-dimensional network [57,58]. Notably, the gel strength of N-HPI in this study surpassed by at least two times that of HPI extracted at pH 11 [12]. As discussed, the high abundance of 11S edestin, the preserved structure, and the relatively lower surface hydrophobicity compared to pH 11-extracted HPI contributed to the higher gel strength of N-HPI. In contrast, R-HPI showed lower gel strength compared to HPI extracted at pH 11, most likely attributed to a relatively lower 11S edistin.

**Figure 2:**
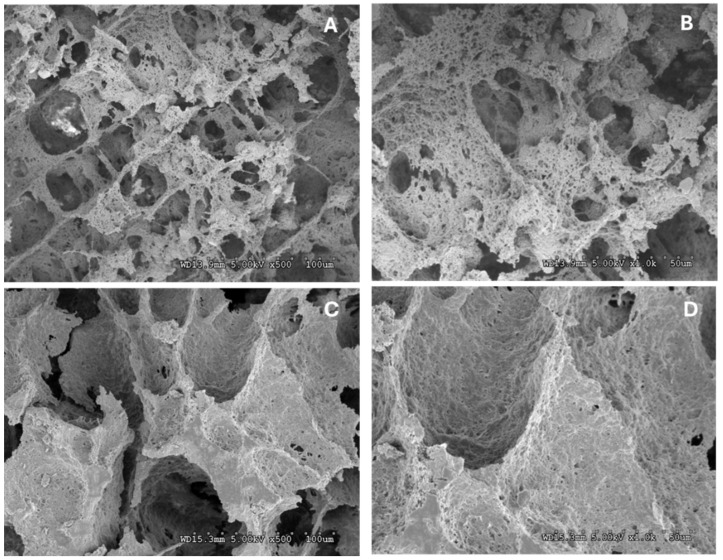
Scanning electron microscopy (SEM) images for NADES-extracted hemp protein isolate (N-HPI) at (**A**) ×500 and (**B**) 1000× magnification, and for reference pH-extracted hemp protein isolate (R-HPI) at (**C**) ×500 and (**D**) 1000× magnification.

SEM images revealed a tighter and denser protein network in N-HPI gels compared to R-HPI counterparts (Figure 2). Moreover, SEM images showed that N-HPI had a more porous structure with interconnected cavities compared to R-HPI. Such a structural pattern may suggest a higher degree of cross-linking and thus stronger gels [59]. This observation is complementary to the gel strength data (Table 4) and thus could also be attributed to the higher ratio of 11 S edestin to 7S vicilin-like protein in N-HPI compared to R-HPI [54]. Due to its high gelling properties, N-HPI can act as a functional replacement for traditional animal-based gelling agents like gelatin, broadening its application in sustainable, non-GMO, allergen-free, gluten-free, and vegan food products.

### 3.5. Effects of NADES-Assisted Extraction on Residual Fat and Off-Flavor Development

#### 3.5.1. Fatty Acid Profile

Hemp seeds have a high oil content and are an excellent source of polyunsaturated fatty acids (PUFAs), such as linoleic, alpha-linolenic, and gamma-linolenic acids [60]. PUFAs are major contributors to off-flavors generated during processing and storage as a result of their oxidation, even when present at low concentrations [61]. Moreover, the presence of lipids in the raw material can lead to the formation of lipid–protein complexes, which can reduce protein extractability and functionality [62,63]. For this reason, reducing the fat content to a minimum is necessary before protein extraction. For R-HPI, defatting was performed using hexane extraction. While for N-HPI, the defatting process was performed simultaneously with protein extraction using two-phase NADES. It was, therefore, imperative to evaluate the impact of these two processes on the fatty acid profile in the hemp protein samples.

**Figure 3:**
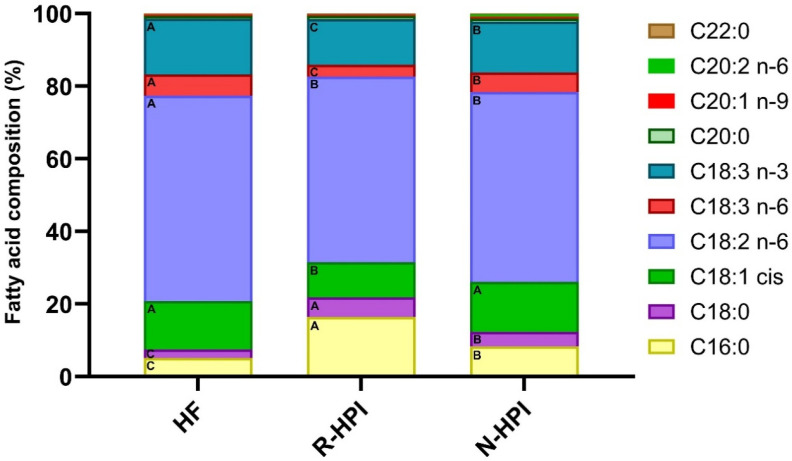
Fatty acid composition in hemp flour (HF), reference pH-extracted and NADES-extracted hemp protein isolate samples (R-HPI, N-HPI). A–C Means (*n* ≥ 3) in each column with different letters are significantly different, according to the Tukey–Kramer multiple means comparison test (*p* < 0.05).

Overall, the detected fatty acids in HF, R-HPI, and N-HPI were similar, except for the long chain 11,14-eicosadienoic acid (C20:2 n-6), which was detected only in N-HPI (Figure 3 and Supplementary Material—Table S3). However, the relative percentages (Figure 3, Supplementary Material Table S3) and actual concentrations (Table S4) of the fatty acids varied among the samples. Linoleic acid (C18:2 n-6) and linolenic acid (C18:3 n-3) were the most abundant PUFA in all samples (Table S3), despite the significant differences in their concentrations among the samples (Table S4). Similarly, linoleic and linolenic acids were reported as the most abundant fatty acids in full-fat hemp flour [64]. Prior to this work, there were no reports on the fatty acid profile of the residual fat in hemp protein isolates.

The use of NADES to simultaneously extract the oil and protein from HF significantly contributed to differences in the residual fatty acid profile of N-HPI compared to R-HPI, which was defatted by hexane prior to protein extraction. For instance, the relative percentage of saturated fatty acids was significantly lower in N-HPI than in R-HPI (Figure 3 and Supplementary Material—Table S3). Conversely, the relative percentage of monounsaturated fatty acid (MUFA), oleic acid, and the relative percentage of PUFAs such as gamma and alfa-linolenic acid were significantly higher in N-HPI in comparison with R-HPI. Additionally, the relative percentage of the major fatty acid, linoleic acid, was not affected by the protein extraction process (Figure 3 and Supplementary Material—Table S3).

However, the absolute concentration of all fatty acids in N-HPI was significantly lower than in the R-HPI (Supplementary Material—Table S4), with the exception of C20:2 n-6. This lower concentration of fatty acids indicated a lower susceptibility to lipid oxidation of N-HPI in comparison to R-HPI. The total fatty acid concentration in N-HPI was about 15-fold lower than that in R-HPI (Table S4), highlighting the efficacy of the two-phase NADES for fat removal, with even better efficiency than defatting with hexane.

While hydrophilic NADES have been extensively used in the agri-food sector [65], hydrophobic NADES were first reported after 2015, and their application in this field is still scarce [17]. However, there are currently no reports on the application of two-phase NADES extraction (a combination of hydrophilic and hydrophobic solvents) in the food sector. This study is the first to utilize a system based on hydrophilic and hydrophobic NADES for simultaneous protein and oil extraction from plant materials. The low concentration of fatty acids in N-HPI highlights the suitability and effectiveness of the one-step NADES-assisted extraction method for the co-extraction of fat and protein. This approach demonstrated superior efficiency of the two-phase NADES extraction compared to hexane defatting, while also providing a more environmentally friendly alternative.

#### 3.5.2. Profile of Volatile Organic Compounds

Off-flavors are the major constraint impacting the acceptance of novel plant proteins [61]. The off-flavors in plant protein ingredients can originate from the starting raw material or can be generated during processing and/or storage [66]. Plant protein ingredients may contain a diverse range of volatile organic compounds (VOCs), which, even at trace concentrations in the parts-per-billion (ppb) or parts-per-million (ppm) range, can prompt a distinct sensory response [61]. Therefore, it is crucial to evaluate how protein extraction processes affect the profile of VOCs to better understand off-flavor formation in HPI.

The evaluation of VOCs in HF, R-HPI, and N-HPI samples revealed a total of 49 VOCs across all samples (Supplementary Material—Table S5). The majority of these VOCs were identified as terpenes and lipid oxidation products, including alcohols, aldehydes, and ketones. Additionally, minor classes such as carboxylic acids, esters, and alkenes were also detected.

**Figure 4:**
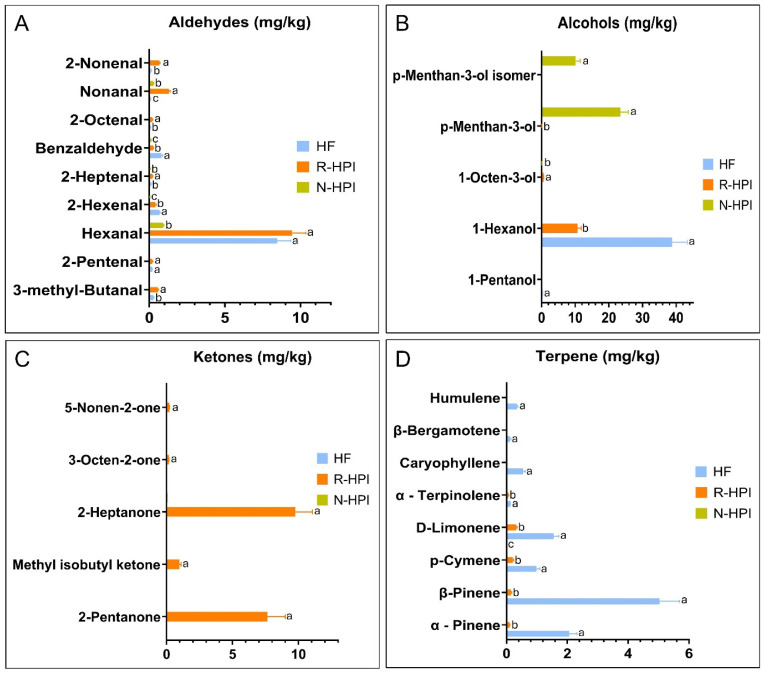
Major aldehydes (**A**), alcohols (**B**), ketones (**C**), and (**D**) terpenes quantified in hemp flour (HF), reference pH-extracted and NADES-extracted hemp protein isolate samples (R-HPI, N-HPI) analyzed by headspace solid-phase microextraction coupled with gas chromatography–mass spectrometry (HS-SPME-GC/MS). (**A**–**C**) Means (*n* ≥ 3) in each compound with different letters are significantly different, according to the Tukey–Kramer multiple means comparison test (*p* < 0.05).

Overall, the primary class of compounds detected in HF were terpenes, alcohols and aldehydes with the highest concentrations for β-pinene, d-limonene, 1-hexanol and hexanal (Supplementary Material—Table S5, Figure 4A,B,D). Terpenes are a large group of volatile phytochemicals that play a key role in mediating both antagonistic and beneficial interactions among organisms. They help protect various species of plants, animals, and microorganisms by defending against predators, pathogens, and competitors [67]. In addition to their characteristic sensory attributes, such as “piney” and “citrusy” odors, terpenes, such as pinene and limonene exhibit notable antimicrobial and antioxidant activities [68]. These compounds were also reported in hemp flowers and were described as bioactive compounds [69]. Although 1-hexanol, an alcohol, is not naturally present in hemp, it can be formed through the oxidation of linoleic acid, the main fatty acid in hemp [70]. This oxidation process can be catalyzed by lipoxygenase, which initially forms hexanal, which is further converted to hexanol by alcohol dehydrogenase [71]. Hexanal was the most abundant aldehyde detected in HF (Figure 4A).

The main VOCs detected in R-HPI were aldehydes and ketones (Figure 4A,C). The main VOCs present in R-HPI were 1-hexanol, hexanal, 2-heptanone, 2-pentanone, and nonanal. The ketones, 2-heptanone and 2-pentanone, can be formed from the degradation of secondary lipid oxidation products originating from PUFA, such as linolenic acid [72]. N-HPI had a significantly lower content of fat-derived VOCs compared to R-HPI (Figure 4A–C). Concentrations lower than 1 mg/kg were detected for hexanal, 2-ethyl-1-hexanol, and nonanal in N-HPI, demonstrating the effectiveness of fat removal using the NADES-assisted extraction process.

**Figure 5:**
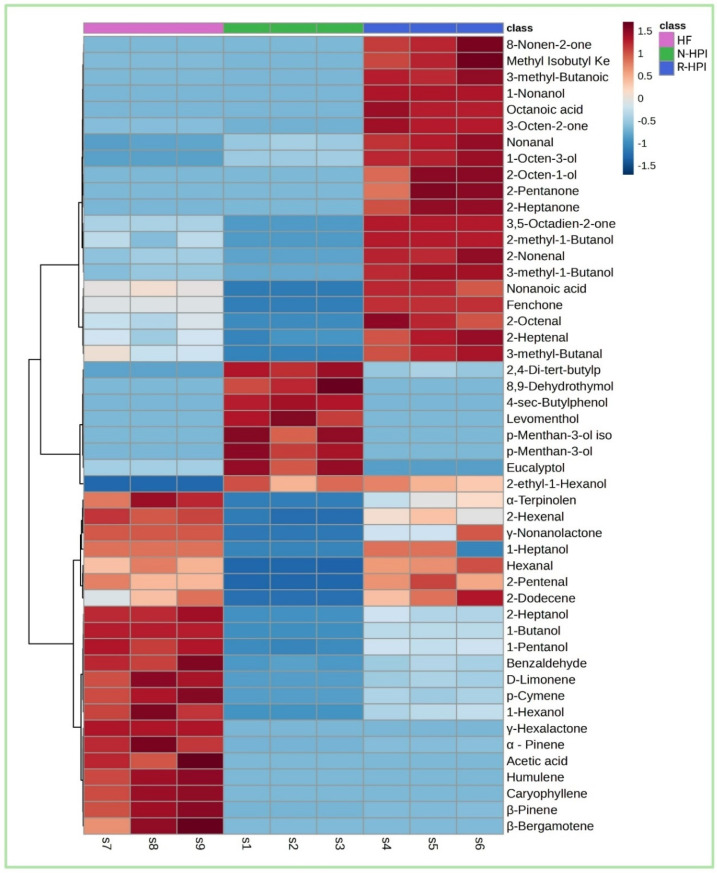
Heatmap illustrating the volatile compound identified in hemp flour (HF), reference pH-extracted and NADES-extracted hemp protein isolate samples (R-HPI, N-HPI).

The differences in the profiles of VOCs among HF, N-HPI, and R-HPI were evaluated using a heatmap (Figure 5). The higher concentration of terpenes and lipid oxidation products found in HF and R-HPI (Figure 5) highlighted the main differences between these samples and N-HPI. In contrast, the profile of VOCs in N-HPI was characterized by the lower concentration of terpenes, such as p-menthan-3-ol, which contributed to its differentiation.

**Figure 6:**
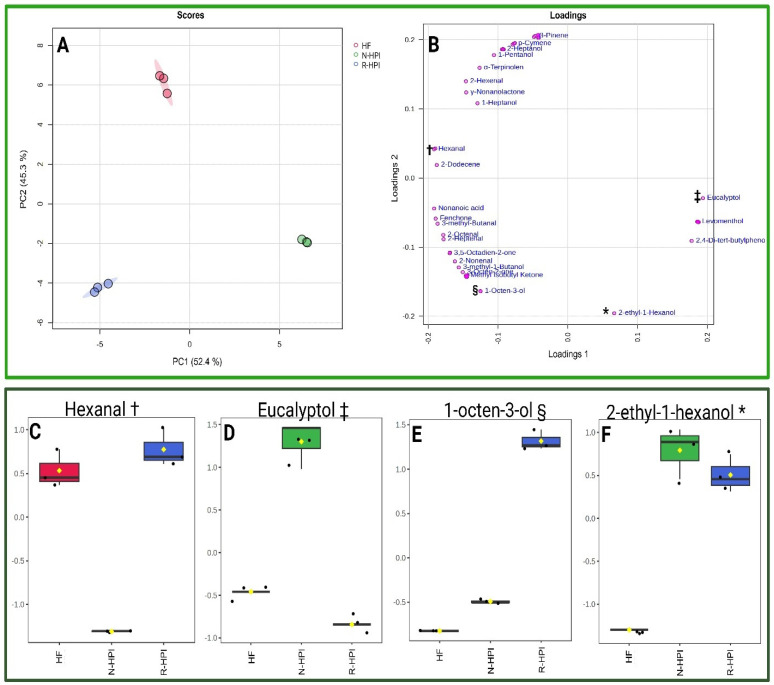
Principal component analysis (PCA) with score plots (**A**) and loadings (**B**). Box-plot with PCA data normalized showing the impact of hexanal (**C**), eucalyptol (**D**), 1-octen-3-ol (**E**), and 2-ethyl-1hexanol (**F**) in the separation of hemp flour (HF), reference pH-extracted and NADES-extracted hemp protein isolate samples (R-HPI, N-HPI). Symbols in the figure were used to highlight the position of the compounds hexanal, eucalyptol, 1-octen-3-ol, and 2-ethyl-1hexanol in the loading plot.

According to PCA (Figure 6A,B) the first and second principal components (PC1 and PC2) explained over 95% of the variance. Hexanal was the primary compound responsible for the separation of the samples along PC1 (Figure 6C,D). Hexanal was the lowest in N-HPI, positioning this sample on the opposite side of the score plot. Conversely, despite their presence in low concentrations, eucalyptol (Figure 6B,E) and 2-ethyl-1-hexanol (Figure 6B,F) were sufficient to discriminate N-HPI on the positive side of PC1. The absence of 2-ethyl-1-hexanol and the lower concentrations of 1-octen-3-olin in HF compared to N-HPI and R-HPI, contributed to the placement of HF on the opposite side of the score plot along PC2. 2-ethyl-1-hexanol and 1-octen-3-ol, which have been previously reported in plant protein isolates [73,74,75], are known to be associated with lipid oxidation processes. Their presence in the HPI samples underscored the occurrence of lipid oxidation during the protein extraction process, highlighting its impact on flavor. The higher concentration of eucalyptol in N-HPI than in HF and R-HPI also contributed to its separation. Eucalyptol, which is a terpene with a mint-like flavor and antimicrobial properties [73], has previously been identified as an important metabolite responsible for differentiating hemp seed varieties [73]. Higher concentrations of eucalyptol in N-HPI than in R-HPI can be associated with selective extraction by the different defatting solvents used.

These observations emphasized that the use of NADES not only effectively reduced the fat content, but also significantly minimized the presence of lipid-derived VOCs, commonly associated with off-flavors, such as hexanal. The reduced presence of these VOCs in N-HPI would potentially contribute to a clean flavor profile, positioning it as a promising protein ingredient for successful incorporation in plant-based formulations, where flavor is a major concern. The ability to reduce off-flavors through strategic extractions like NADES-assisted extraction can broaden the applications of hemp protein in food systems, enhancing consumer acceptance and expanding its use in plant-based food products.

## 4. Conclusions

This study demonstrated the effectiveness of a novel two-phase NADES-assisted extraction process for the simultaneous defatting and extraction of hemp protein, without the use of hazardous solvents. As such, the study addressed the long-standing challenges of conventional defatted and protein extraction processes. By eliminating the need for organic solvents like hexane and employing mild protein extraction conditions, the two-phase NADES-assisted extraction not only can preserve the structural and functional integrity of the extracted protein but also has the potential to promote sustainable processes. Compared to the HPI produced by hexane defatting followed by conventional alkaline protein extraction, N-HPI achieved significantly higher protein yield and purity, superior solubility under acidic conditions, and superior gelation properties. Furthermore, the relatively low VOCs in N-HPI could contribute to a clean flavor protein ingredient, positioning hemp protein as a promising ingredient for successful incorporation in plant-based formulations. This work is a significant step forward in the development of sustainable extraction technologies, showcasing a practical pathway for reducing the environmental footprint of plant protein production, while addressing key consumer demands for clean-label ingredients. Future research directions could include scaling up the NADES-assisted extraction process to assess its industrial feasibility and conducting a detailed life cycle assessment to evaluate its environmental footprint compared to conventional methods. A techno-economic analysis would also be crucial to determine the cost-effectiveness and economic viability of implementing this technology at an industrial scale. Furthermore, the adaptability of this method could be explored with other underutilized or emerging oilseeds, such as camelina, sunflower, or flaxseed, to broaden its applicability and impact. This work serves as a foundation for future studies aiming to optimize sustainable extraction technologies and expand the portfolio of high-quality plant protein ingredients for food innovation. Such a development will pave the way for integrating hemp protein into a wide range of food products, supporting sustainable and health-conscious food innovations.

## 5. Patents

Dias, F. F. G., & Ismail, B. P. (2024) [76]. Provisional Patent: Tunable Solvents and Methods of Use. Provisional Patent UMN 2024-286-MRG 0110.000757US60.

## Figures and Tables

**Table 1:** Protein extraction purity (%) and yield (%) of hemp protein isolates (HPIs), pellet, and gum fractions as affected by the composition of solvent during NADES-assisted extraction, as well as ash and fat content (%) of each HPI sample.

Trial	Amount (g) of ChCl: Glycerol + Menthol: Thymol ^1^	HPI				Discarded Pellet ^2^		Discarded Gums ^3^	
		Protein Purity ^4^ (%)	Protein Yield ^5^ (%)	Ash (%)	Fat (%)	Protein Purity ^4^ (%)	Protein Residue ^6^ (%)	Protein Purity ^4^ (%)	Protein Lost ^7^ (%)
1	100 + 0	88.2 ^b^	75.2 ^b^	1.02 ^a^	2.94 ^a^	16.4 ^a^	23.5 ^a^	13.1 ^b^	2.20 ^b^
2	100 + 5	94.0 ^a^	81.7 ^ab^	1.05 ^a^	1.18 ^b^	13.1 ^b^	8.80 ^b^	11.3 ^b^	4.41 ^a^
3	100 + 15	89.8 ^b^	83.3 ^a^	1.02 ^a^	0.920 ^b^	17.5 ^a^	8.65 ^b^	20.8 ^a^	3.66 ^ab^

^1^ Chlorine chloride in glycerol (hydrophilic solvent) plus menthol in thymol (hydrophobic solvent); ^2^ Pellet discarded after NADES-assisted extraction; ^3^ Gums discarded after NADES solubilization; ^4^ Protein purity (%) represents the amount of protein in the freeze-dried sample determined by the Dumas method; ^5^ Protein yield (%) represents the amount of protein extracted relative to the total amount of protein in the starting hemp flour; ^6^ Protein residue (%) represents the amount of protein left in the discarded pellet relative to the total amount of protein in the starting hemp flour; ^7^ Protein lost (%) represents the amount of protein lost to the discarded gum relative to the total amount of protein in the starting hemp flour; ^a^^–^^b^ Means (*n* = 3) in each column with different lowercase letters are significantly different, according to the Tukey–Kramer multiple means comparison test (*p* < 0.05).

**Table 2:** Color (L* a* b*) of commercial soy and pea protein isolate (cSPI, cPPI), reference pH-extracted and NADES-extracted hemp protein isolate samples (R-HPI, N-HPI).

Sample	L*	a*	b*
cSPI	74.8 ^b^	−4.47 ^a^	14.9 ^c^
cPPI	72.1 ^c^	−3.31 ^b^	18.1 ^a^
R-HPI	67.0 ^d^	−2.74 ^c^	16.9 ^b^
N-HPI	77.2 ^a^	−4.31 ^a^	10.7 ^d^

^a–d^ Means in each column with different lowercase letters are significantly different, according to the Tukey–Kramer multiple means comparison test (*p* < 0.05).

**Table 3:** Surface hydrophobicity, surface charge, denaturation temperature and enthalpy of commercial soy and pea protein isolate (cSPI, cPPI), reference pH-extracted and NADES-extracted hemp protein isolate samples (R-HPI, N-HPI).

Samples	Surface Properties			Denaturation Temperature and Enthalpy	
	Surface Hydrophobicity (RFI)	Surface Charge (mV)		Denaturation Temperature (Td, °C)	Enthalpy of Denaturation (ΔH, Jg^−1^)
		pH 7.0	pH 3.4		
cSPI	7570 ^c^	−42.9 ^a^	29.8 ^b^	~ ^1^	~
cPPI	13,500 ^b^	−34.3 ^b^	25.9 ^c^	~	~
R-HPI	16,700 ^a^	−27.1 ^c^	32.4 ^b^	83.9	7.63
N-HPI	15,400 ^a^	−22.4 ^d^	40.9 ^a^	84.2	14.9 *

^1^ ~No peak of denaturation was observed; ^a^^–^^d^ Means (*n* ≥ 3) in each column with different lowercase letters are significantly different, according to the Tukey–Kramer multiple means comparison test (*p* < 0.05); * An asterisk indicates significant difference in denaturation between N-HPI and R-HPI as tested by the independent samples *t*-test (*p* < 0.05).

**Table 4:** Solubility, gel strength, and emulsification capacity of commercial soy and pea protein isolate (cSPI, cPPI), reference pH-extracted and NADES-extracted hemp protein isolate samples (R-HPI, N-HPI).

Sample	Protein Solubility				Emulsification Capacity (g oil/g protein)	Gel Strength Strength ^1^ (N)
	pH 7.0		pH 3.4			
	Non-Heated	Heated at 80 °C	Non-Heated	Heated at 80 °C		
cSPI	62.2 ^a^	77.5 ^a^	10.8 ^c^	18.4 ^c^	1090 ^a^	26.9 ^b^
cPPI	18.3 ^c^	32.0 ^b^	9.80 ^c^	16.8 ^c^	577 ^b^	7.71 ^d^
R-HPI	26.8 ^b^	25.3 ^c^	53.2 ^b^	54.4 ^b^	583 ^b^	11.1 ^c^
N-HPI	6.18 ^d^	6.21 ^d^	92.5 ^a^	93.8 ^a^	282 ^c^	57.7 ^a^

^1^ cSPI, N-HPI, and R-HPI protein solutions were prepared at 15% protein (w/v), while cPPI protein solution was prepared at 20% protein (w/v); ^a^^–^^d^ Means (*n* ≥ 3) in each column with different lowercase letters are significantly different, according to the Tukey–Kramer multiple means comparison test (*p* < 0.05).

## Data Availability

Data is contained within the article or Supplementary Material.

## Supplementary Materials

The following supporting information can be downloaded at: https://www.mdpi.com/article/10.3390/plants14020274/s1. Table S1. Protein extraction purity (%) and yield for hemp protein isolate (HPI) samples and pellet fractions at different extraction pHs (7 to 11) and ash content (%) of HPI. Table S2. Protein solubility of pH-extracted (pH 7–11) hemp protein isolate (HPI) samples measured at 5% protein concentration at both pH 7.0 and 3.4 with and without heating. Table S3. Fatty acid profile (%) in hemp flour (HF), reference pH-extracted, and NADES-extracted hemp protein isolate samples (R-HPI, N-HPI). Table S4. Concentration of fatty acid (ug/g of sample) in hemp flour (HF), reference pH-extracted, and NADES-extracted hemp protein isolate samples (R-HPI, N-HPI). Table S5. Volatiles in hemp flour (HF), reference pH-extracted, and NADES-extracted hemp protein isolate samples (R-HPI, N-HPI).

## References

[1] Plant-Based Protein Market Size & Forecast [Latest]. https://www.marketsandmarkets.com/Market-Reports/plant-based-protein-market-14715651.html.

[2] Wild F., Czerny M., Janssen A., Kole A., Zunabovic M., Domig K. (2014). The Evolution of a Plant-Based Alternative to Meat: From Niche Markets to Widely Accepted Meat Alternatives. Agro Food Ind. Hi-Tech.

[3] Naghshi S., Sadeghi O., Willett W.C., Esmaillzadeh A. (2020). Dietary Intake of Total, Animal, and Plant Proteins and Risk of All Cause, Cardiovascular, and Cancer Mortality: Systematic Review and Dose-Response Meta-Analysis of Prospective Cohort Studies. BMJ.

[4] Ismail B.P., Senaratne-Lenagala L., Stube A., Brackenridge A. (2020). Protein Demand: Review of Plant and Animal Proteins Used in Alternative Protein Product Development and Production. Anim. Front..

[5] Xiao X., Zou P.-R., Hu F., Zhu W., Wei Z.-J. (2023). Updates on Plant-Based Protein Products as an Alternative to Animal Protein: Technology, Properties, and Their Health Benefits. Molecules.

[6] Rizzo G., Storz M.A., Calapai G. (2023). The Role of Hemp (*Cannabis sativa* L.) as a Functional Food in Vegetarian Nutrition. Foods.

[7] Mark T.B., Shepherd J., Olson D., Snell W., Proper S., Thornsbury S. (2020). Economic Viability of Industrial Hemp in the United States: A Review of State Pilot Programs.

[8] (2018). GRAS Notice No. GRN 000771, Seed (GRN 000765), Hemp Seed Protein Powder (GRN 000771), and Hemp Seed Oil (GRN 000778). https://www.fda.gov/media/164016/download.

[9] Baldino N., Carnevale I., Mileti O., Aiello D., Lupi F.R., Napoli A., Gabriele D. (2022). Hemp Seed Oil Extraction and Stable Emulsion Formulation with Hemp Protein Isolates. Appl. Sci..

[10] Kumar S.P.J., Prasad S.R., Banerjee R., Agarwal D.K., Kulkarni K.S., Ramesh K.V. (2017). Green Solvents and Technologies for Oil Extraction from Oilseeds. Chem. Cent. J..

[11] Lazarjani M.P., Young O., Kebede L., Seyfoddin A. (2021). Processing and Extraction Methods of Medicinal Cannabis: A Narrative Review. J. Cannabis Res..

[12] Eckhardt L., Bu F., Franczyk A., Michaels T., Ismail B.P. (2024). Hemp (*Cannabis sativa* L.) Protein: Impact of Extraction Method and Cultivar on Structure, Function, and Nutritional Quality. Curr. Res. Food Sci..

[13] (2005). Toxicological Review of n-Hexane.

[14] Gao Z., Shen P., Lan Y., Cui L., Ohm J.-B., Chen B., Rao J. (2020). Effect of Alkaline Extraction pH on Structure Properties, Solubility, and Beany Flavor of Yellow Pea Protein Isolate. Food Res. Int..

[15] Tang J., Yao D., Xia S., Cheong L., Tu M. (2024). Recent Progress in Plant-Based Proteins: From Extraction and Modification Methods to Applications in the Food Industry. Food Chem. X.

[16] Mišan A., Pojić M., Verpoorte R., Witkamp G.-J., Choi Y.H. (2021). Chapter Thirteen—Applications of NADES in Stabilizing Food and Protecting Food Compounds against Oxidation. Advances in Botanical Research.

[17] Mišan A., Nađpal J., Stupar A., Pojić M., Mandić A., Verpoorte R., Choi Y.H. (2020). The Perspectives of Natural Deep Eutectic Solvents in Agri-Food Sector. Crit. Rev. Food Sci. Nutr..

[18] Karimi A., Bhowmik P., Yang T.C., Samaranayaka A., Chen L. (2024). Extraction of Canola Protein via Natural Deep Eutectic Solvents Compared to Alkaline Treatments: Isolate Characteristics and Protein Structural and Functional Properties. Food Hydrocoll..

[19] Guzmán-Lorite M., Marina M.L., García M.C. (2022). Successive Extraction Using Natural Deep Eutectic Solvents and Pressurized Liquids for a Greener and Holistic Recovery of Proteins from Pomegranate Seeds. Food Res. Int..

[20] Parodi E., La Nasa J., Ribechini E., Petri A., Piccolo O. (2023). Extraction of Proteins and Residual Oil from Flax (*Linum usitatissimum*), Camelina (*Camelina sativa*), and Sunflower (*Helianthus annuus*) Oilseed Press Cakes. Biomass Convers. Biorefinery.

[21] Choi Y.H., van Spronsen J., Dai Y., Verberne M., Hollmann F., Arends I.W.C.E., Witkamp G.J., Verpoorte R. (2011). Are natural deep eutectic solvents the missing link in understanding cellular metabolism and physiology?. Plant Physiol..

[22] Dai Y., van Spronsen J., Witkamp G.-J., Verpoorte R., Choi Y.H. (2013). Natural deep eutectic solvents as new potential media for green technology. Anal. Chim. Acta.

[23] Smith E.L., Abbott A.P., Ryder K.S. (2020). Deep eutectic solvents (DESs) and their applications. Chem. Rev..

[24] Dias F.F.G., De Almeida N.M., De Souza T.S.P., Taha A.Y., De Moura Bell J.M.L.N. (2020). Characterization and Demulsification of the Oil-Rich Emulsion from the Aqueous Extraction Process of Almond Flour. Processes.

[25] Machida K., Huang Y.-P., Furlan Gonçalves Dias F., Barile D., Leite Nobrega de Moura Bell J.M. (2022). Leveraging Bioprocessing Strategies to Achieve the Simultaneous Extraction of Full-Fat Chickpea Flour Macronutrients and Enhance Protein and Carbohydrate Functionality. Food Bioprocess Technol..

[26] Almeida F.S., Dias F.F.G., Sato A.C.K., de Moura Bell J.M.L.N. (2022). Scaling up the Two-Stage Countercurrent Extraction of Oil and Protein from Green Coffee Beans: Impact of Proteolysis on Extractability, Protein Functionality, and Oil Recovery. Food Bioprocess Technol..

[27] Dias F.F.G., Taha A.Y., de Moura Bell J.L.N. (2022). Effects of Enzymatic Extraction on the Simultaneous Extraction of Oil and Protein from Full-Fat Almond Flour, Insoluble Microstructure, Emulsion Stability and Functionality. Future Foods.

[28] de Almeida N.M., Dias F.F.G., Rodrigues M.I., de Moura Bell J.M.L.N. (2019). Effects of Processing Conditions on the Simultaneous Extraction and Distribution of Oil and Protein from Almond Flour. Processes.

[29] Thirulogasundar A., Shi D., Stone A.K., Xu C., Bhagwat A., Lu Y., Polley B., House J.D., Bhowmik P., Rajagopalan N. (2024). Effect of Enzyme Hydrolysis on the Functionality, Protein Quality, and Flavour Profile of Lentil and Chickpea Protein Isolates. J. Food Meas. Charact..

[30] Gasparre N., Rosell C.M., Boukid F. (2024). Enzymatic Hydrolysis of Plant Proteins: Tailoring Characteristics, Enhancing Functionality, and Expanding Applications in the Food Industry. Food Bioprocess Technol..

[31] FoodData Central. https://fdc.nal.usda.gov/fdc-app.html#/food-details/170148/nutrients.

[32] Latimer G.W., AOAC International, Association of Official Analytical Chemists International (2023). Official Methods of Analysis of AOAC International: 3-Volume Set.

[33] AACC International (1999). Method 44-40.01: Moisture—Modified Vacuum-Oven Method. AACC Approved Methods of Analysis.

[34] Boyle C., Hansen L., Hinnenkamp C., Ismail B. (2018). Emerging Camelina Protein: Extraction, Modification, and Structural/Functional Characterization. J. Am. Oil Chem. Soc..

[35] Bu F., Nayak G., Bruggeman P., Annor G., Ismail B.P. (2022). Impact of Plasma Reactive Species on the Structure and Functionality of Pea Protein Isolate. Food Chem..

[36] Dias F.F.G., Augusto-Obara T.R., Hennebelle M., Chantieng S., Ozturk G., Taha A.Y., Vieira T.M.F.d.S., de Moura Bell J.M.L.N. (2020). Effects of Industrial Heat Treatments on Bovine Milk Oxylipins and Conventional Markers of Lipid Oxidation. Prostaglandins Leukot. Essent. Fat. Acids.

[37] Oliveira W.S., Chen Q., Edleman D., Annor G.A., Dias F.F.G. (2024). Unraveling the Impacts of Germination on the Volatile and Fatty Acid Profile of Intermediate Wheatgrass (*Thinopyrum intermedium*) Seeds. Molecules.

[38] Xiao L., Lee J., Zhang G., Ebeler S.E., Wickramasinghe N., Seiber J., Mitchell A.E. (2014). HS-SPME GC/MS Characterization of Volatiles in Raw and Dry-Roasted Almonds (*Prunus dulcis*). Food Chem..

[39] Cui L., Kimmel J., Zhou L., Rao J., Chen B. (2020). Identification of Extraction pH and Cultivar Associated Aromatic Compound Changes in Spray Dried Pea Protein Isolate Using Untargeted and Targeted Metabolomic Approaches. J. Agric. Food. Res..

[40] Stone A.K., Karalash A., Tyler R.T., Warkentin T.D., Nickerson M.T. (2015). Functional Attributes of Pea Protein Isolates Prepared Using Different Extraction Methods and Cultivars. Food Res. Int..

[41] Paredes-López O., Ordorica-Falomir C., Olivares-Vázquez M.R. (1991). Chickpea Protein Isolates: Physicochemical, Functional and Nutritional Characterization. J. Food Sci..

[42] Kumar M., Tomar M., Potkule J., Verma R., Punia S., Mahapatra A., Belwal T., Dahuja A., Joshi S., Berwal M.K. (2021). Advances in thePlant Protein Extraction: Mechanism and Recommendations. Food Hydrocoll..

[43] Zhang Z., He S., Liu H., Sun X., Ye Y., Cao X., Wu Z., Sun H. (2020). Effect of pH Regulation on the Components and Functional Properties of Proteins Isolated from Cold-Pressed Rapeseed Meal through Alkaline Extraction and Acid Precipitation. Food Chem..

[44] Hewage A., Olatunde O.O., Nimalaratne C., House J.D., Aluko R.E., Bandara N. (2024). Improved Protein Extraction Technology Using Deep Eutectic Solvent System for Producing High Purity Fava Bean Protein Isolates at Mild Conditions. Food Hydrocoll..

[45] Sze-Tao K.W.C., Sathe S.K. (2000). Functional Properties and in Vitro Digestibility of Almond (*Prunus dulcis* L.) Protein Isolate. Food Chem..

[46] Dias F.F.G., de Moura Bell J.M.L.N. (2022). Understanding the Impact of Enzyme-Assisted Aqueous Extraction on the Structural, Physicochemical, and Functional Properties of Protein Extracts from Full-Fat Almond Flour. Food Hydrocoll..

[47] Husband H., Ferreira S., Bu F., Feyzi S., Ismail B.P. (2024). Pea Protein Globulins: Does Their Relative Ratio Matter?. Food Hydrocoll..

[48] Hadnađev M., Dapčević-Hadnađev T., Lazaridou A., Moschakis T., Michaelidou A.-M., Popović S., Biliaderis C.G. (2018). Hempseed Meal Protein Isolates Prepared by Different Isolation Techniques. Part I. Physicochemical Properties. Food Hydrocoll..

[49] Feige M.J., Braakman I., Hendershot L.M. (2018). Disulfide Bonds in Protein Folding and Stability. Oxidative Folding of Proteins: Basic Principles, Cellular Regulation and Engineering.

[50] Hansen L., Bu F., Ismail B.P. (2022). Structure-Function Guided Extraction and Scale-Up of Pea Protein Isolate Production. Foods.

[51] Yaputri B.P., Bu F., Ismail B.P. (2023). Salt Solubilization Coupled with Membrane Filtration-Impact on the Structure/Function of Chickpea Compared to Pea Protein. Foods.

[52] Ajibola C.F., Aluko R.E. (2022). Physicochemical and Functional Properties of 2S, 7S, and 11S Enriched Hemp Seed Protein Fractions. Molecules.

[53] McClements D.J. (2004). Food Emulsions: Principles, Practices, and Techniques.

[54] Dapčević-Hadnađev T., Dizdar M., Pojić M., Krstonošić V., Zychowski L.M., Hadnađev M. (2019). Emulsifying Properties of Hemp Proteins: Effect of Isolation Technique. Food Hydrocoll..

[55] Lam R.S.H., Nickerson M.T. (2013). Food Proteins: A Review on Their Emulsifying Properties Using a Structure–Function Approach. Food Chem..

[56] Karaca A.C., Low N., Nickerson M. (2011). Emulsifying Properties of Chickpea, Faba Bean, Lentil and Pea Proteins Produced by Isoelectric Precipitation and Salt Extraction. Food Res. Int..

[57] Totosaus A., Montejano J.G., Salazar J.A., Guerrero I. (2002). A Review of Physical and Chemical Protein-Gel Induction. Int. J. Food Sci. Technol..

[58] Nakai S. (1983). Structure-Function Relationships of Food Proteins: With an Emphasis on the Importance of Protein Hydrophobicity. J. Agric. Food Chem..

[59] Sharma S., Majumdar R.K., Mehta N.K. (2022). Gelling Properties and Microstructure of the Silver Carp Surimi Treated with Pomegranate (*Punica granatum* L.) Peel Extract. J. Food Sci. Technol..

[60] Karabulut G., Kahraman O., Pandalaneni K., Kapoor R., Feng H. (2023). A Comprehensive Review on Hempseed Protein: Production, Functional and Nutritional Properties, Novel Modification Methods, Applications, and Limitations. Int. J. Biol. Macromol..

[61] Damodaran S., Arora A. (2013). Off-Flavor Precursors in Soy Protein Isolate and Novel Strategies for Their Removal. Annu. Rev. Food Sci. Technol..

[62] Chen H., Xu B., Wang Y., Li W., He D., Zhang Y., Zhang X., Xing X. (2023). Emerging Natural Hemp Seed Proteins and Their Functions for Nutraceutical Applications. Food Sci. Hum. Wellness.

[63] Yue J., Gu Z., Zhu Z., Yi J., Ohm J.-B., Chen B., Rao J. (2021). Impact of Defatting Treatment and Oat Varieties on Structural, Functional Properties, and Aromatic Profile of Oat Protein. Food Hydrocoll..

[64] Banskota A.H., Jones A., Hui J.P.M., Stefanova R. (2022). Triacylglycerols and Other Lipids Profiling of Hemp By-Products. Molecules.

[65] Katrak V.K., Ijardar S.P. (2024). Redefining the Landscape of Protein Extraction and Separation from Various Sources Using Deep Eutectic Solvents. Trends Food Sci. Technol..

[66] Oliveira W.d.S., Shepelev I., Dias F.F.G., Reineccius G.A. (2024). Advances in Sample Preparation for Volatile Profiling of Plant Proteins: Fundamentals and Future Perspectives. Adv. Sample Prep..

[67] Dudareva N., Klempien A., Muhlemann J.K., Kaplan I. (2013). Biosynthesis, Function and Metabolic Engineering of Plant Volatile Organic Compounds. New Phytol..

[68] Chen C., Pan Z. (2021). Cannabidiol and Terpenes from Hemp—Ingredients for Future Foods and Processing Technologies. J. Future Foods.

[69] Brkljača N., Đurović S., Milošević S., Gašić U., Panković D., Zeković Z., Pavlić B. (2023). Sequential extraction approach for sustainable recovery of various hemp (*Cannabis sativa* L.) bioactive compounds. Sustain. Chem. Pharm..

[70] Xu J., Xu X., Yuan Z., Hua D., Yan Y., Bai M., Song H., Yang L., Zhu D., Liu J. (2022). Effect of Hemp Protein on the Physicochemical Properties and Flavor Components of Plant-Based Yogurt. LWT.

[71] Espino-Díaz M., Sepúlveda D.R., González-Aguilar G., Olivas G.I. (2016). Biochemistry of Apple Aroma: A Review. Food Technol. Biotechnol..

[72] Grebenteuch S., Kanzler C., Klaußnitzer S., Kroh L.W., Rohn S. (2021). The Formation of Methyl Ketones during Lipid Oxidation at Elevated Temperatures. Molecules.

[73] Song W., Yin H., Zhong Y., Wang D., Xu W., Deng Y. (2022). Regional Differentiation Based on Volatile Compounds via HS-SPME/GC–MS and Chemical Compositions Comparison of Hemp (*Cannabis sativa* L.) Seeds. Food Res. Int..

[74] Xu M., Jin Z., Gu Z., Rao J., Chen B. (2020). Changes in Odor Characteristics of Pulse Protein Isolates from Germinated Chickpea, Lentil, and Yellow Pea: Role of Lipoxygenase and Free Radicals. Food Chem..

[75] Murat C., Bard M.-H., Dhalleine C., Cayot N. (2013). Characterisation of Odour Active Compounds along Extraction Process from Pea Flour to Pea Protein Extract. Food Res. Int..

[76] Dias F.F.G., Ismail B.P. (2024). Tunable Solvents and Methods of Use. Provisional Patent.

