# Supplementary material for: Impact of a Novel Two-Phase Natural Deep Eutectic Solvent-Assisted Extraction on the Structural, Functional, and Flavor Properties of Hemp Protein Isolates
Source: Plants (Basel). 2025 Jan 18;14(2):274. doi: 10.3390/plants14020274 (PMC11768760; doi:10.3390/plants14020274)
Supplement: Supplementary file 1 [file plants-14-00274-s001.zip › plants-3403746-supplementary.pdf]

Supplementary material

Impact of a novel two-phase natural deep eutectic solvent-assisted extraction on the structural, functional, and flavor properties of hemp protein

Yi Chen, Wellington Oliveira, Fernanda F. G. Dias*, B. Pam Ismail*

Department of Food Science and Nutrition, University of Minnesota, St. Paul, MN 55108, USA

Table 1S. Protein extraction purity (%) and yield for hemp protein isolate (HPI) samples and pellet fractions at different extraction pHs (7 to 11) and ash content (%) of HPI.

Solubilization pH	HPI			Discarded Pellet[1]		Discarded Supernatant[2]	
	Protein Purity[3] (%)	Protein Yield[4] (%)	Ash (%)	Protein Purity[3] (%)	Protein Residue[5] (%)	Protein Purity[3] (%)	Protein Lost[6] (%)
7	73.4[d]	3.91[e]	2.55[bc]	76.8[a]	86.3[a]	27.8[bc]	6.85[a]
8	76.3[c]	8.10[d]	2.60[ab]	75.0[b]	82.4[b]	28.5[b]	6.22[ab]
9	84.4[b]	23.4[c]	2.63[ab]	71.1[c]	67.7[c]	30.5[a]	7.10[a]
10	90.5[a]	63.8[b]	2.37[c]	49.7[d]	26.6[d]	28.8[b]	6.46[ab]
11	91.1[a]	88.6[a]	2.78[a]	2.99[e]	0.780[e]	26.4[c]	5.76[b]

[1]Pellet discarded after alkaline solubilization;
[2]Supernantant discarded after isoelectric point precipitation;
[3]Protein purity (%) represents the amount of protein in the freeze-dried sample determined by the Dumas method;
[4]Protein yield (%) represents the amount of protein extracted relative to the total amount of protein in the starting defatted hemp meal (DHM);
[5]Protein residue (%) represents the amount of protein left in the discarded pellet relative to the total amount of protein in the starting DHM;
[6]Protein lost (%) represents the amount of protein lost to the discarded supernatant relative to the total amount of protein in the starting DHM;
[a-e] Means (n = 3) in each column with different lowercase letters are significantly different, according to the Tukey-Kramer multiple means comparison test ($P < 0.05$).

Table 2S. Protein solubility of pH-extracted (pH 7-11) hemp protein isolate (HPI) samples measured at 5% protein concentration at both pH 7.0 and 3.4 with and without heating.

pH of protein extraction	% Solubility			
	pH 7.0		pH 3.4	
	Non-heated	Heated at 80 °C	Non-heated	Heated at 80 °C
11	4.23^{e}	4.90^{d}	53.5^{c}	56.1^{b}
10	6.01^{d}	6.12^{d}	67.9^{a}	67.7^{a}
9	13.9^{c}	12.5^{c}	65.5^{a}	65.2^{a}
8	23.9^{b}	23.4^{b}	52.2^{c}	53.8^{b}
7	48.7^{a}	46.1^{a}	57.6^{b}	55.0^{b}

$^{a\text{-}e}$ Means in each column with different lowercase letters are significantly different, according to the Tukey-Kramer multiple means comparison test (P <0.05).

Table 3S. Fatty acid profile (%) in hemp flour (HF), reference pH-extracted, and NADES-extracted hemp protein isolate samples (R-HPI, N-HPI).

Fatty Acid		HF	R-HPI	N-HPI
Palmitic acid	C16:0	5.12[c]	16.3[a]	8.27[b]
Stearic acid	C18:0	2.33[c]	5.41[a]	3.99[b]
Oleic acid	C18:1 cis	13.3[a]	9.70[b]	13.8[a]
Linoleic acid	C18:2 n-6	56.6[a]	51.1[b]	52.3[b]
Gamma-linolenic acid	C18:3 n-6	5.83[a]	3.33[c]	5.41[b]
Linolenic acid	C18:3 n-3	15.3[a]	12.5[c]	14.0[b]
Eicosanoic acid	C20:0	0.840[c]	0.930[a]	0.890[ab]
Gondoic Acid	C20:1 n-9	0.340[b]	0.310[b]	0.510[a]
11,14-Eicosadienoic acid	C20:2 n-6	ND[1]	ND	0.550[a]
Behenic acid	C22:0	0.310[b]	0.360[b]	0.330[ab]

[1]ND: non detected;

[a-c] Means ($n \geq 3$) in each raw, within each fatty acid and across the different samples, with different lowercase letters are significantly different, according to the Tukey-Kramer multiple means comparison test ($P < 0.05$).

Table 4S. Concentration of fatty acid (ug/g of sample) in hemp flour (HF), reference pH-extracted, and NADES-extracted hemp protein isolate samples (R-HPI, N-HPI).

Fatty Acid		HF	R-HPI	N-HPI
Palmitic acid	C16:0	13,410[a]	7,835[b]	261.9[c]
Stearic acid	C18:0	6,107[a]	2,593[b]	126.3[c]
Oleic acid	C18:1 cis	34,860[a]	4,645[b]	435.9[c]
Linoleic acid	C18:2 n-6	148,100[a]	24,470[b]	1653[c]
Gamma-linolenic acid	C18:3 n-6	15,270[a]	1,596[b]	170.5[c]
Linolenic acid	C18:3 n-3	40,120[a]	5982[b]	442.80[c]
Eicosanoic acid	C20:0	2192[a]	446.3[b]	28.12[c]
Gondoic Acid	C20:1 n-9	883.6[a]	150.5[b]	15.74[c]
11,14-Eicosadienoic acid	C20:2 n-6	ND[1]	ND	17.39[a]
Behenic acid	C22:0	800.3[a]	170.5[b]	10.34[b]
	Total	261,740	47,890	3,162

[1]ND: non detected;

[a-c] Means (n ≥ 3) in each raw, within each fatty acid and across the different samples, with different lowercase letters are significantly different, according to the Tukey-Kramer multiple means comparison test (P <0.05).

Table 5S. Volatiles in hemp flour (HF), reference pH-extracted, and NADES-extracted hemp protein isolate samples (R-HPI, N-HPI).

Nº	RT	Compound	Match (%)	RICal	RITab	Δ	Concentration (ppb)		
							HF	R-HPI	N-HPI
1	2.14	Acetic acid	94.8	685	660	25	874.5[a]	ND[1]	1.563[b]
2	2.27	3-methyl-Butanal	84.6	693	669	24	265.9[b]	572.1[a]	ND
3	2.32	1-butanol	89.3	696	669	27	57.74[a]	15.91[b]	ND
4	2.52	2-pentanone	98.1	709	715	-6	ND	7639[a]	ND
5	3.12	3-methyl-1-butanol	94.8	747	741	6	26.87[b]	250.90[a]	1.072[b]
6	3.16	2-methyl-1-butanol	80.6	750	736	14	15.79[b]	72.76[a]	6.001[b]
7	3.16	Methyl isobutyl ketone	94.2	750	733	17	11.00[b]	965.20[a]	ND
8	3.41	2-pentenal	82.4	766	755	11	180.0[a]	197.7[a]	13.02[b]
9	3.61	1-pentanol	92.1	778	780	-2	319.4[a]	120.0[b]	5.144[c]
10	4.23	Hexanal	97.5	817	817	0	8460[a]	9452[a]	930.80[b]
11	5.26	3-methyl-butanoic acid	82.1	858	834	24	10.86[b]	3220[a]	ND
12	5.44	2-hexenal	88.1	865	854	11	668.0[a]	397.3[b]	20.01[c]
13	5.88	1-hexanol	98.8	882	880	2	38770[a]	10730[b]	39.69[c]
14	6.41	2-heptanone	84.3	903	898	5	23.36[b]	9767[a]	83.52[b]
15	6.76	2-heptanol	83.3	917	901	16	132.2[a]	43.60[b]	ND
16	7.75	α - pinene	97.2	947	939	8	2057[a]	96.17[b]	ND
17	8.51	2-heptenal	85	970	978	-8	104.3[b]	209.80[a]	55.04[c]
18	8.65	Benzaldehyde	86.5	974	965	9	802.8[a]	248.9[b]	103.5[c]
19	8.96	1-heptanol	85.4	984	975	9	11.94[a]	5.904[b]	ND
20	9.31	1-octen-3-ol	82.2	994	986	8	12.76[c]	612.80[a]	103.20[b]
21	9.61	β-pinene	92.7	1003	984	19	5031.0[a]	149.40[b]	18.97[c]
22	10.83	p-cymene	93.2	1038	1030	8	985.60[a]	209.80[b]	21.260[c]
23	10.98	2-ethyl-1-Hexanol	95.6	1042	1029	13	ND	154.04[a]	173.06[a]
24	10.99	D-limonene	96	1043	1032	11	1551.0[a]	316.70[b]	26.70[c]
25	11.09	Eucalyptol	81.4	1045	1036	9	11.50[b]	ND	47.82[a]

Table 5S. Continued.

Nº	RT	Compound	Match (%)	RICal	RITab	Δ	HF	R-HPI	N-HPI
26	11.31	3-octen-2-one	89.1	1052	1046	6	13.08[b]	171.73[a]	0.7179[c]
27	11.75	γ-Hexalactone	88.1	1064	1055	9	19.33[a]	ND	ND
28	12.04	2-octenal	90.8	1072	1050	22	81.20[b]	197.60[a]	22.02[c]
29	12.36	2-octen-1-ol	95.8	1081	1067	14	ND	37.41[a]	ND
30	12.42	3,5-octadien-2-one	83.9	1083	1093	-10	14.20[b]	42.61[a]	0.376[c]
31	12.84	8-nonen-2-one	86.3	1094	1080	14	ND	205.80[a]	ND
32	12.99	α-terpinolene	88	1099	1089	10	118.50[a]	59.77[b]	ND
33	13.12	Fenchone	83	1102	1088	14	9.24[b]	20.70[a]	ND
34	13.73	Nonanal	96.4	1119	1108	11	82.12[c]	1289[a]	266.9[b]
35	15.70	2-nonenal	96.6	1173	1171	2	110.1[b]	659.1[a]	ND
36	16.09	Octanoic acid	85.5	1184	1182	2	ND	363.4[a]	ND
37	16.14	1-nonanol	85.3	1185	1172	13	ND	11.22[a]	ND
38	16.33	p-menthan-3-ol	98.7	1191	1173	18	ND	35.44[b]	23440[a]
39	16.53	p-Menthan-3-ol isomer	99.6	1196	1170	26	1.301[b]	2.201[b]	10101[a]
40	16.76	Levomenthol	95.6	1202	1188	14	ND	ND	109.0[a]
41	16.85	2-dodecene	86.5	1205	1205	0	38.98[a]	50.36[a]	ND
42	17.70	8,9-dehydrothymol	95	1229	1221	8	ND	ND	371.93[a]
43	19.44	Nonanoic acid	81.1	1279	1280	-1	114.3[b]	203.0[a]	ND
44	21.45	4-sec-Butylphenol	91.2	1338	1315	23	ND	ND	29780[a]
45	22.51	γ-Nonanolactone	85.4	1369	1368	1	21.78[a]	13.96[b]	ND
46	24.46	Caryophyllene	97.7	1429	1419	10	554.94[a]	ND	ND
47	24.91	β-bergamotene	81.4	1443	1436	7	110.3[a]	2.351[b]	ND
48	25.59	Humulene	92.2	1465	1457	8	336.10[a]	ND	ND
49	27.20	2,4-di-tert-butylphenol	97.9	1516	1512	4	ND	32.60[b]	193.4[a]

Nº: Compound number; RT: Retention time; RICal: retention index calculated; RITab: Retention index reported in National Institute of Standards and Technology; Δ: variation of Ris.

[1] ND: non detected;

[a-c] Means ($n \geq 3$) in each raw, within each fatty acid and across the different samples, with different lowercase letters are significantly different, according to the Tukey-Kramer multiple means comparison test ($P < 0.05$).
